# A single nucleotide polymorphism assay sheds light on the extent and distribution of genetic diversity, population structure and functional basis of key traits in cultivated north American cannabis

**DOI:** 10.1186/s42238-020-00036-y

**Published:** 2020-09-11

**Authors:** Philippe Henry, Surender Khatodia, Karan Kapoor, Britni Gonzales, Alexis Middleton, Kevin Hong, Aaron Hilyard, Steve Johnson, Davis Allen, Zachary Chester, Dan Jin, José Carlos Rodriguez Jule, Iain Wilson, Manu Gangola, Jason Broome, Deron Caplan, Dinesh Adhikary, Michael K. Deyholos, Michael Morgan, Oliver W. Hall, Brent J. Guppy, Cindy Orser

**Affiliations:** 1 VSSL Enterprises Ltd., West Kelowna, BC Canada; 2 Digipath Labs Inc., Las Vegas, NV USA; 3 Island Genetics Ltd., Vancouver, BC Canada; 4 Biomedical Engineering, University of Alberta, Edmonton, AB Canada; 5 Polar Bear Genome BioPharma, Edmonton, AB Canada; 6 Labs-Mart Inc., Edmonton, AB Canada; 7 Okanagan Gold Cannabis Corp, West Kelowna, BC Canada; 8 The Flowr Group (Okanagan) Inc., Kelowna, BC Canada; 9 HYTN Beverages, Vancouver, BC Canada; 10 Biology, The University of British Columbia Okanagan, Kelowna, BC Canada; 11 Noble Growth Corp, Drayton Valley, AB Canada; 12 Synthase Genetics Inc., Winnipeg, MB Canada; 13 OneLeaf Cannabis Co., Regina, Saskatchewan Canada; 14 Botanist Organic Growers, Winnipeg, Manitoba Canada

**Keywords:** Cannabis, Hemp, Genetic assay, Cannabinoids, Terpenes, Compliance, Population structure

## Abstract

**Background:**

The taxonomic classification of Cannabis genus has been delineated through three main types: *sativa* (tall and less branched plant with long and narrow leaves), *indica* (short and highly branched plant with broader leaves) and *ruderalis* (heirloom type with short stature, less branching and small thick leaves). While still under discussion, particularly whether the genus is polytypic or monotypic, this broad classification reflects putative geographical origins of each group and putative chemotype and pharmacologic effect.

**Methods:**

Here we describe a thorough investigation of cannabis accessions using a set of 23 highly informative and polymorphic SNP (Single Nucleotide Polymorphism) markers associated with important traits such as cannabinoid and terpenoid expression as well as fibre and resin production. The assay offers insight into cannabis population structure, phylogenetic relationship, population genetics and correlation to secondary metabolite concentrations. We demonstrate the utility of the assay for rapid, repeatable and cost-efficient genotyping of commercial and industrial cannabis accessions for use in product traceability, breeding programs, regulatory compliance and consumer education.

**Results:**

We identified 5 clusters in the sample set, including industrial hemp (K5) and resin hemp, which likely underwent a bottleneck to stabilize cannabidiolic acid (CBDA) accumulation (K2, Type II & III). Tetrahydrocannabinolic acid (THCA) resin (Type I) makes up the other three clusters with terpinolene (K4 - colloquial “sativa” or “Narrow Leaflet Drug” (NLD), myrcene/pinene (K1) and myrcene/limonene/linalool (K3 - colloquial “indica”, “Broad Leaflet Drug” (BLD), which also putatively harbour an active version of the cannabichrometic acid Synthase gene (CBCAS).

**Conclusion:**

The final chemical compositions of cannabis products have key traits related to their genetic identities. Our analyses in the context of the NCBI *Cannabis sativa* Annotation Release 100 allows for hypothesis testing with regards to secondary metabolite production. Genetic markers related to secondary metabolite production will be important in many sectors of the cannabis marketplace. For example, markers related to THC production will be important for adaptable and compliant large-scale seed production under the new US Domestic Hemp Production Program.

## Background

Cannabis, an annual and dioecious member of the family Cannabaceae, is an economically important genus providing protein- and oil-rich seeds, long and short fibres for industrial applications (construction materials, textiles, paper, etc.), and a wide diversity of secondary metabolites found predominantly as terpenoids and cannabinoids (Lynch et al. 2016; McPartland 2018; Onofri and Mandolino 2017). In fact, the cannabis plant can produce over 150 unique terpenoids and roughly 100 unique cannabinoids, with a subset showing bona fide therapeutic utility (Hanuš et al. 2016; Booth and Bohlmann 2019). However, despite the large diversity in secondary metabolite profiles across thousands of cultivars, the stratification into drug-type cannabis or fibre-type cannabis hinges on the dry weight concentration of a single cannabinoid, Δ^9^-tetrahydrocannabinol (THC). This approach which prevails today in the USDA interim regulations, employs a THC concentration of 0.3% as the threshold separating hemp and drug-type cultivars, with concentrations below 0.3% defined as hemp (Dolgin 2019). Other jurisdictions outside North America have adopted higher thresholds, for example in Switzerland where concentration under 1% total THC is considered a compliant hemp crop. Sadly, despite human cultivation for over 6000 years in varying climates worldwide (Clarke and Merlin 2013), its evolution, taxonomic classification, and phylogenetic connections remain poorly understood. These deficiencies stem from limited research, irregular breeding efforts, unorganized selection, ex situ conservation, and government restrictions, which ultimately resulted in the high heterozygosity observed within cannabis genomes today (e.g. Rahn et al. 2016; McPartland 2018).

Although a subject of ongoing debate, taxonomic classification of the Cannabis genus has been delineated through three main types: 1) *sativa* (tall and less branched plant with long and narrow leaves), 2) *indica* (short and highly branched plant with broader leaves) and 3) *ruderalis* (heirloom with short stature, less branching and small thick leaves). While still under debate, particularly whether the genus is polytypic or monotypic, this broad classification reflects the putative geographical origins of each group (Clarke and Merlin 2013; Lynch et al. 2016, Schwabe and McGlaughlin 2019). Consequently, there is currently no structured horticultural registration system available for cannabis cultivars (varieties), instead these are often awarded the epithet “strains”, which are likely the outcome of extensive hybridization and subsequent rehybridization from their original botanical descriptors (Henry 2015).

The recent legalization of drug type cannabis for commercial production and recreational use in Canada, several US States, and other countries worldwide, as well a hemp under the US 2018 Farm Bill has created renewed scientific interest in developing a robust empirical classification system for cannabis. To that end, a particular focus has been placed on secondary metabolite expression with a clear separation based on CBD (cannabidiol):THC ratios. Differences in CBD:THC ratios delineate three class types: type-I (ratio < 0.5), type-II (ratio 0.5–3.0) and type-III (ratio > 3.0) (Elzinga et al. 2015), Interestingly, a genetic basis for these types can be determined by polymorphism at the CBDAS and THCAS genes on Chromosome 9 (Laverty et al. 2019). In addition, double recessives at this locus give rise to type-IV (cannabigerolic acid, CBGA accumulators; de Meijer and Hammond 2005) whereas type-V plants are free of cannabinoids which may have resulted from functionally ablative mutations founds within the upstream components of the cannabinoid synthase pathway (de Meijer et al. 2009). More recently, the addition of terpenoids as potential chemotaxonomic markers have emerged as a preferred model compared to cannabinoids alone (e.g. Lewis et al. 2018). Linking chemotype to genotype has also enabled deeper insight into a novel consumer-centric classification based on genetic markers associated with chemical expression (e.g. Orser and Henry 2019). Recently, others have proposed targeted markers for the identification of fiber and resin cannabis (e.g. Cascini et al. 2019; Hilyard et al. 2019) as well as molecular sexing tools to differentiate feminized from regular seed stock (Toth et al. 2020).

In addition to paving the way for empirical taxonomic classification, genetic information can provide insight into the extent and distribution of genetic variability, population structure, phylogenetic relationships, as well as providing the essential tools required to perform marker assisted selection in order to improved homozygosity and trait stability. In addition, genetic information can faithfully identify matching multilocus genotypes across disparate accessions. This feature may be particularly useful in seed-to-sale tracking as it provides an irrefutable identity for each individual accession and paves the way for cannabis variety registration and protection.

Here we describe a thorough investigation of cannabis accessions using a set of 23 highly informative and polymorphic SNP markers associated with important traits such as cannabinoid and terpenoid expression (Henry 2017; Henry et al. 2018; Orser and Henry 2019). We extend the scope of sampling to 420 accessions from Licenced Cultivators in Saskatchewan, Manitoba, British Columbia, Canada as well as Nevada, USA. We validate the use of these 23 SNP markers to assess population structure, phylogenetic relationship, population genetics, and correlation to secondary metabolite concentrations, and demonstrate the utility of this assay for rapid, repeatable and cost efficient genotyping of commercial and industrial cannabis accessions for use in product traceability, breeding programs, and consumer education.

## Methods

### Sample collection

Samples were collected reflecting the diversity of cannabis germplasm available in North America, with samples from industrial hemp lines (type-III), resin hemp (type-II and type-III) and THC drug-type (type-I) cannabis. The sampling strategy did not follow any particular selection criteria as samples were randomly chosen from several licenced cultivators who graciously agreed to have their accessions analysed in this study (Supplementary Table S3). Given the sensitivity of our genotyping approach, a small 2mm^2^ segment of leaf tissue was collected at each facility and was sufficient to yield adequate DNA for downstream genotyping.

### DNA isolation procedure

Prior to performing the DNA extraction protocol, and in order to obtain high molecular weight DNA, plant tissue samples were lyophilised by allowing to air dry for 24-48 h at room temperature and in the presence of silica desiccant. Lyophilised plant tissue was homogenised in a 1.5 ml microcentrifuge tube with a reusable pestle. Homogenised material was then treated following the Sbeadex® plant mini kit protocol (LGC Biosearch Technologies, Beverley, MA) following the manufacturer’s instructions. After the addition of 90 μL Lysis buffer PN, samples were incubated at 65 °C for > 10 min. The samples were then centrifuged at 2500 x g for 10 min to pellet the debris. 50 μL of the supernatant in this tube, referred to as the lysate, was then transferred to a clean 1.5 ml microcentrifuge tube with 120 μL Binding buffer PN and 10 μL Sbeadex® particle suspension and incubated at room temperature for 4 min. The tube was then brought into contact with a magnet for roughly 1 min until magnetic particles form a pellet. The supernatant was then discarded, and the pellet was then subjected to three consecutive wash steps. The washed beads were then eluted with 70 μL Elution buffer PN and incubated at 55 °C for 3 min prior to bringing the tubes in contact with the magnet. 50 μL of the eluate was then transferred to a new tube which contain high purity plant DNA.

### Endpoint PCR genotyping using custom KASP assays

Twenty-three optimized assay mixes, each specific to single nucleotide polymorphisms (SNP) previously identified as associated with phylogeny and chemotypic expression were screened in the sample set (Henry 2015, 2017; Henry et al. 2018). These assays consist of two competitive, allele-specific forward primers and one common reverse primer (KASP; LGC Biosearch Technologies, Beverley, MA). Each forward primer incorporates an additional tail sequence that corresponds with one of two universal FRET (fluorescent resonance energy transfer) cassettes present in the KASP Master mix which contains the two FRET cassettes (FAM and HEX), ROX™ passive reference dye, Taq polymerase, free nucleotides and MgCl_2_ in an optimised buffer solution.

The genotypes were generated using an Eco RT (Illumina, San Diego, CA), a CFX 96 (Biorad, Hercules, CA) and an Intelliqube array tape platform (LGC Biosearch Technologies, Beverley, MA) with multiple blind replicates across platforms to ensure cross system repeatability. Genotypes were called using the Kluster Caller software and manually verified using the SNPviewer software (LGC Biosearch Technologies, Beverley, MA).

### Functional basis of 23 SNP

Given the recent release of the 10-chromosome map of the cannabis genome (Grassa et al. 2018; Laverty et al. 2019), metabolomic and proteomic insight (Jenkins and Orsburn 2019a, 2019b) as well as a fully annotated version of the cannabis genome resulting from the completion of the NCBI *Cannabis sativa* Annotation Release 100 (Jenkins and Orsburn 2019c), we set out to characterise the functional basis of the SNPs used in the study. The previously designed targets developed using Cansat 3 (van Bakel et al. 2011) were subjected to a BLASTn search (Altschul et al. 1990) constrained to the taxa cannabis using the NCBI online interface (https://blast.ncbi.nlm.nih.gov) accessed October 31, 2019. The location of the 10-chromosome map as well as the putative functional gene in which the 23 SNP are found were recorded.

### Statistical analyses of genotypic data

Multilocus genotypes were formatted as a table (comma separated file) of genotypes with individuals as rows and markers as columns. As the total dataset of 681 plant DNA samples contained some missing data, we culled all missing data out and undertook the following analyses on 420 samples with complete genotype information across all markers. Metadata, including individual and population names, were separated from the genotype data and imported into the flexible statistical environment of R (R Core Team 2018) requiring the following packages, *ape* (Paradis and Schliep 2018), *pegas* (Paradis 2010), *poppr* (Kamvar et al. 2014), *adegenet* (Jombart 2008) and *hierfstat* (Goudet and Jombart 2015).

Briefly, the *read.loci* function was used to import the allelic data into the R environment as a data frame which was then converted to a *genind* object using the *df2genind* command. Individual and population (variety identity) were also incorporated into the *genind* object to allow for population level calculations to shed light on the stability of claimed variety names and to assess the level of genetic diversity within and between these hypothesized groups. Clonal lines were identified using *mlg* and *mlg.id* functions, which determines the number and identity of mutilocus genotypes. Basic population genetics metrics, particularly expected heterozygosity was calculated for each population and individual using the *poppr* function.

To shed light on the underlying relationships between our diverse sample set, a dissimilarity matrix or Hamming distance between multilocus genotypes was calculated using the *bitwise.dist* function and was visualized using a phylogenetic tree using the *nj* function. Principal component analyses (PCA) were undertaken to provide an independent line of evidence of the genetic affinities between accessions using the *dudi.pca* function. Broad signals of population genetic structure were investigated using discriminant analyses of principal components (DAPC; Jombart 2008). The optimal number of clusters was determined using the *find.cluster* function followed by the *dapc* function using said clusters as the most likely observed structure. The DAPC was visualized using the *scatter* function. A minimum spanning tree calculated from the squared distance between individual was plotted to shed light on the phylogenetic relationship of each inferred cluster. Lastly, the inferred clusters were applied as the population factor and the genetic differentiation between populations (variety names) as well as for the inferred clusters were calculated using the *pairwise.fst* function. Diversity indices for varieties representing putative seed lines for which at least three individuals were available in the dataset were also assessed using the *locus_ table* function where variety names were used as population indicator.

### Statistical analyses of chemotypic data

A subset of 120 samples from Nevada were also analyzed by various LC / MS combination at 9 cannabinoid and 17 terpenes, following the methods described by Orser et al. (2018). Since the genetic panel was developed to find the most informative genetic markers associated with chemotypic expression, we grouped individuals according to the clusters from the DAPC and visualized the chemotype variation using side by side boxplots of the top cannabinoid and monoterpenes. Similarly, R was used to read the chemotypic data using the *read.table* function. The *boxplot* function was used to plot the top cannabinoid and terpenes expressed in each cluster.

## Results

### Extent and distribution of genetic diversity and population structure in modern Cannabis

**Fig. 1:**
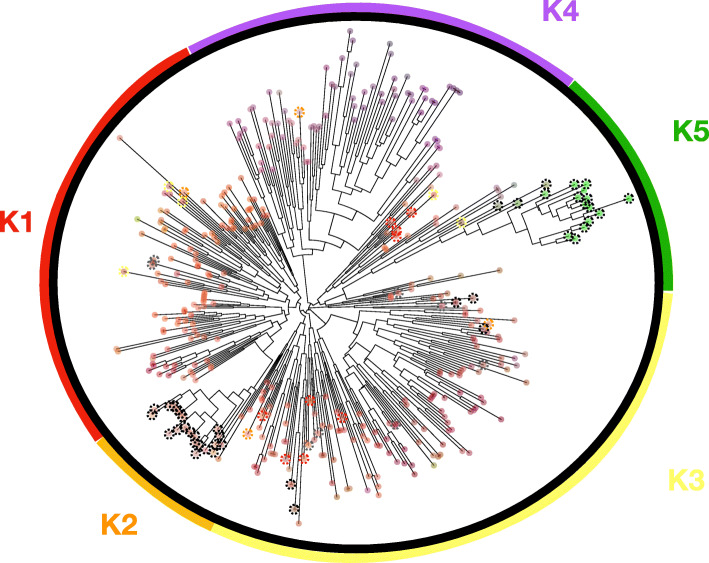
Neighbour-joining tree. Showing the relative phylogenetic location of the 420 cannabis accessions typed at 23 single nucleotide polymorphisms (SNP). Discriminant analysis of principal component (DAPC) clusters are shown with K1-K5 represented by different colors. K1-K4 are resin type cannabis and K5 is the fiber type cannabis or hemp. Colored dotted circles highlight individuals assigned differently between the neighbor-joining tree and DAPC clusters. Type-III plants are shown with a dotted black circle and type-II plants are shown with dotted grey circle

**Fig. 2:**
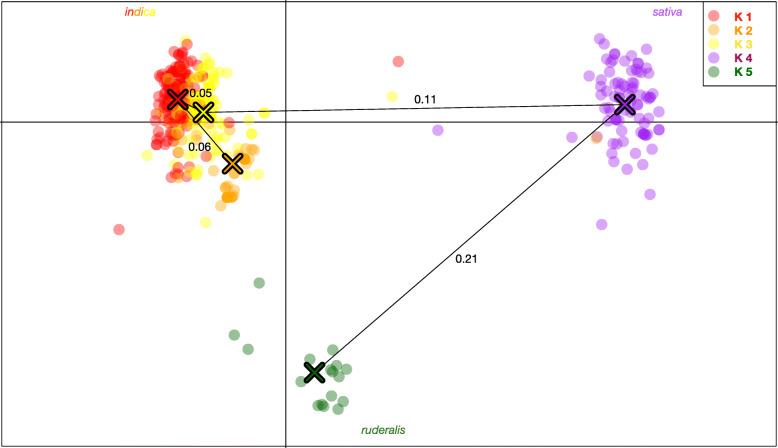
Discriminant analysis of principal component (DAPC) scatterplot. Showing the relative location of each individual sample in two dimensional space, overlaid by a minimum spanning tree calculated from the squared distance between individual to represent the phylogenetic relationship between inferred clusters. K5, hemp or “*ruderalis*” appears ancestral and the most differentiated group, followed by K4, terpinolene dominant resin accessions. The genetic distance between groups (*Fst*) is indicated on the respective branches of the minimum spanning tree

**Fig. 3:**
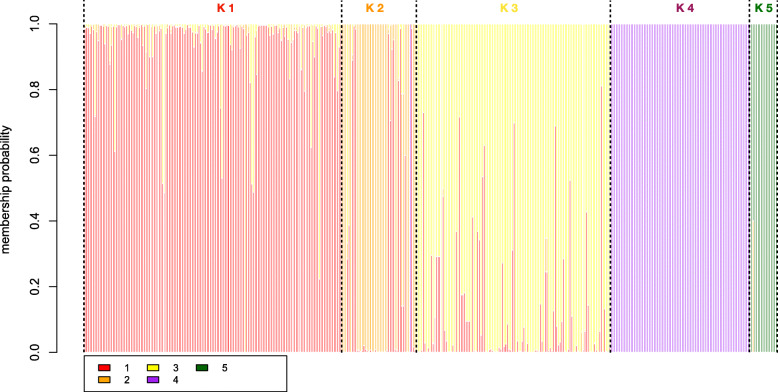
Discriminant analysis of principal component (DAPC) genotype composition plot. Showing the membership probability of each individual’s (columns) assignment of genotype to each clusters K1 – K5. Mis-assigned individuals can easily be identified as well as F1 hybrids with mixed genotypes

The 23 SNP panel used in this study was selected to represent a broad coverage of the cannabis genome and individual SNPs were found to be located on all cannabis linkage groups with the exception of chromosome 8 (Table 1). As such, levels of polymorphism varied widely between SNPs, from fixed mitochondrial alleles that allow for the discrimination between fibre-type and resin-type cannabis (Figs. 1, 2 and 3), to highly variable nuclear markers. Of note, two resin-type landrace varieties from Kyrgyzstan and Egypt were the exception to the rule, both displaying the fibre-type mitochondrial haplotype while expressing THC as the main cannabinoid.

**Table 1:** Statistics, population genetic metrics and main chemotypes for inferred clusters K1-K5

K	N	MLG	H	Hexp	Ia	Terp1	Terp 2	Terp 3	Canna
**1**	156	135	4.8	0.31	0.22	Myrcene	Limonene	Linalool	THCA CBCA
**2**	45	30	3.1	0.31	1.40	p-Cymene	Carene		CBDA CBCA
**3**	118	104	4.6	0.29	0.21	Myrcene	a-pinene		THCA CBCA
**1–3**	319	269	5.5	0.32	0.34	Myrcene	a-pinene	Limonene	THCA CBDA CBCA
**4**	84	75	4.3	0.26	0.26	Terpenolene	Ocimene	Caryophyllene	THCA CBGA
**5**	17	17	2.8	0.133	1.12	Hemp			CBDA
**Total**	420	361	5.8	0.33	0.57				

K- Cluster

N - Number of individual samples per population

MLG - the number of multilocus genotypes found in the specified population

H - Shannon-Weiner Diversity index

Hexp - Nei’s gene diversity (expected heterozygosity)

Ia - Index of Association for each population factor

Terp - Terpene

Canna - Cannabinoid

THCA - Tetrahydrocannabinolic acid

CBDA - Cannabidiolic acid

CBGA - Cannabigerolic acid

CBCA - Cannabichrometic acid

Heterozygosity at the nuclear markers ranged from 0.03 to 0.50 (Table 1, Supplementary Table S1). Three markers targeting the THCAS gene cluster offered strong discrimination of major cannabis groups, associated with the two major pentyl cannabinoids THC and CBD. In particular, the *SW6 and VSSL_BtBD* markers were fixed for one allele in all CBD expressing varieties (fibre and resin-types), while being fixed for other allele or heterozygote in all THC expressing varieties. In addition, the SVIP14 locus was also strongly associated with cannabinoid expression data (Table 2).

**Table 2:** Information about the 23 SNPs used in the study. Including genomic location and putative function. Bolded markers indicate those with significant association to the inferred population structure described here

Assays ID	SNP	Chromosome	Location	Gene
**SVIP5**	T/A	5	69,688,051 - 69,692,326	*Cannabis sativa* uncharacterized LOC115717933
**SW6**	G/A	9	25,821,934 - 25,823,723	**Inactive THCAS / CBCAS**
**SVIP9**	A/G	5	82,096,056 - 82,098,831	Cannabis sativa uncharacterized LOC115718065
**SVIP10**	C/T	4	10,679,414 - 10,682,584	Cannabis sativa neurofilament medium polypeptide-like
**SVIP13**	A/G	2	10,112,681 - 10,121,235	Cannabis sativa uncharacterized LOC115705170
**SVIP14**	A/T	3	417,333 - 420,067	**Cannabis sativa bifunctional endo-1,4-beta-xylanase XylA-like (LOC115710019),** **mRNA**
**SVIP15**	G/A	10	59,112,921 - 59,117,320	Cannabis sativa ribose-phosphate pyrophosphokinase 4
**SVIP16**	A/C	10	60,829,569 - 60,837,666	Cannabis sativa probable leucine-rich repeat receptor-like protein kinase At1g35710
**SVIP19**	A/G	1	71,846,233 - 71,850,866	Cannabis sativa mechanosensitive ion channel protein 8-like
**SVIP21**	G/A	10	58,184,100 - 58,188,483	Cannabis sativa uncharacterized membrane protein At3g27390
**SVIP22**	A/G	4	88,963,964 - 88,967,267	Cannabis sativa solute carrier family 35 member F5
**SVIP23**	C/T	10	47,593,480 - 47,598,098	Cannabis sativa Cs2S genes for albumin
**VSSL_BtBd**	C/T	9	25,821,934 - 25,823,723	**Inactive THCAS / CBCAS**
**VSSL_A250D**	C/A	9	25,821,934 - 25,823,723	**Inactive THCAS / CBCAS**
**VSSL_mito**	C/A	Mitochondria	317,914 - 318,214	**Downstream of trnC tRNA**
**VSSL_digi2**	C/A	5	14,237,657 - 14,252,007	**Cannabis sativa O-glucosyltransferase rumi homolog**
**VSSL_digi3**	T/C	6	27,445,636 - 27,447,293	Cannabis sativa uncharacterized LOC115719990
**VSSL_digi4**	T/A	10	56,459,661 - 56,460,726	Cannabis sativa uncharacterized LOC115700304
**VSSL_digi6**	C/T	7	1,868,696 - 1,880,067	Cannabis sativa transcriptional corepressor LEUNIG_HOMOLOG
**VSSL_digi7**	G/A	6	74,036,351 - 74,039,762	Cannabis sativa pentatricopeptide repeat-containing protein At5g59600 (LOC115718943), transcript variant X2
**VSSL_digi12**	T/C	5	37,063,921 - 37,071,583	Cannabis sativa K(+) efflux antiporter 5-like
**VSSL_digi14**	C/T	3	211,984 - 216,544	**Cannabis sativa putative disease resistance RPP13-like protein 1**
**VSSL_digi19**	G/A	7	56,524,106 - 56,525,416	Cannabis sativa uncharacterized LOC115722935

**Fig. 4:**
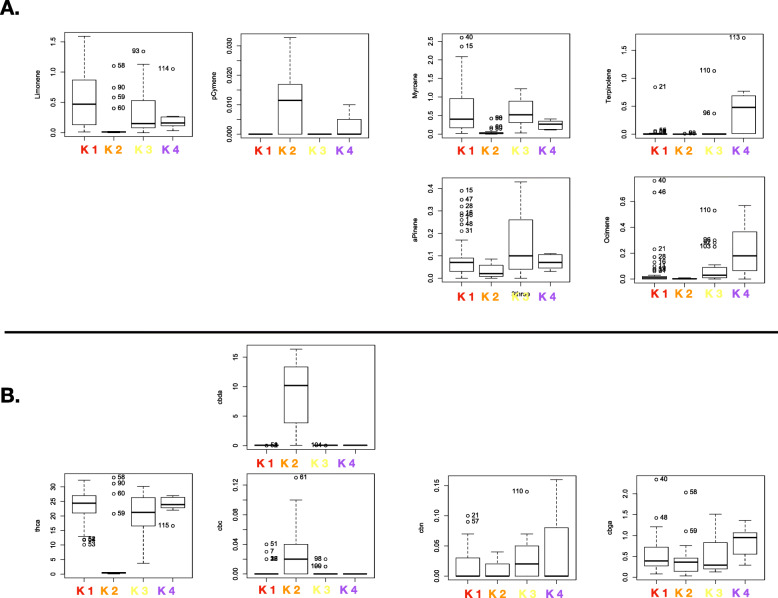
Boxplots of chemotypic data for each inferred K1- K4. No chemotype data was available for K5, yet all individuals from that cluster are expected to display a low resin type-III phenotype. **a** Total terpene percentage per dry weight content as determined by GC-MS. **b** Total cannabinoid percentage per dry weight content as determined by HPLC

The DAPC exercise clustered cannabis varieties into five groups (Figs. 1, 2 and 3), which was mostly congruent with the independent neighbor joining tree (Fig. 1). European Hemp (K5; 15 individuals, C. s. *ruderalis*, typically fibre or grain cultivars, often non- photoperiodic) was clearly distinct from all drug-type cannabis accessions, including high CBD resin expressing accessions. Interestingly resin (drug)-type cannabis consisted of four main genetic clusters, K1 and 3 (156, 118 individuals, myrcene/pinene, myrcene/limonene/linalool dominant respectively) which can be considered having a *C. s. indica* phenotype and perceived effect, while K4 (84 individuals, terpinolene) contain mainly accessions of equatorial or *C. s. sativa* designation. K2 (45 individuals, cymene dominant) consisted mostly of the high CBD resin-hemp from the United States (Fig. 4).

One known first generation hybrid (“S2”) between an autoflowering male “*Darryl*” and a CBD resin-type named “*Intergallactic Princess*” (not sampled here) was found to be assigned to both K2 and K5 in a 40:60 proportion skewed towards the father’s origin (Fig. 3). Other possible F1 hybrids were detected between K1 and K3 as well as possibly mis-assigned THC resin individuals into the K2 cluster (Figs. 1, 2, 3).

### Multilocus genotypes, identification of identical clones

In total, 361 multilocus genotypes (putative clonal lines) were identified in the 420 cannabis samples. Fourteen pairs of known labelled clones were confirmed using the 23 SNP assay. Mislabelled accessions with identical multilocus genotypes were frequently detected as follows: “*Unidentified*” and “*Hindu Kush*”, “*GGC*” and “*Purple God*”, “*Atomical Haze*” and “*Tangerine Dream*” and “SFVOG”, “*Gorilla Glue*” and “*Holy Grail*”, “*Agent Orange*” and “*Girl Scout Cookies*”, “*UK Cheese*” and “*Jamaican Ten Speed*”, “*Chem 91*” and “*Colorado Sunset*”*,* “*Jet Fuel*” *and* “*Louis VIII*”*,* “*Blackberry Cream*” and “*Slime Dawg MillaNaire*”*,* “*Tangerine Dream*” and “*Violator Kush*”, “*Original Amnesia*” and “*Sour Tangie*”*,* “*Billy Crystal*” and “*Blueberry Kush*”*,* “*5th Dimension*” *and* “*Gorilla Glue*”*,* “*Garlic*” *and* “*Gelato Breath*”*,* “*Blue Dream*” with two “*Blue Hash Plant*” samples, seven samples including five labelled “*Pink Kush*”, one mislabelled “*Atomical Haze*” and one *“LA Lights”,* seven unlabelled Resin Hemp from Nevada, including one labeled *“Cherry Wine”,* as well as three Resin Hemp samples labelled “Alamo”, “Adam” and “Shore”.

### Diversity within seed lines and inferred clusters

Twenty of the 23 markers were found to deviate from Hardy-Weinberg equilibrium (HWE) in at least one of the 71 populations/seed lines (Supplementary Figure S1), which was not surprising in itself, given the domestication history and strong selective forces for chemical expression in modern North American commercial cannabis cultivars. Of interest when repeated in the larger clusters determined using DAPC, a total of four markers were found to not deviate from HWE (Supplementary Figure S2). The average heterozygosity within seed lines (putative populations) was 0.33, which was considered much higher than what was to be expected in any other major stable commercial crops. Interestingly, the most homozygous line, with heterozygosity of 0.09 was the Canadian fiber/grain cultivar “*X59*” (Supplementary Material Table S1, Table S2). Several drug cultivars, including “*Pink Kush*”, “*Punch Breath*”, “*Durga Matta II CBD*”, “*Durga Matta*”, “*Cotton Candy*”, “*Chem4OG*”, “*33rd Degree”* and “*ASD*” all from known seed banks displayed relatively good stability with heterozygosities below 0.2. Another metric of interest is the index of association (*Ia*; Brown et al. 1980). This index brings an additional insight as a tool to quantify the reshuffling of alleles that occurs in sexually outcrossing species. A deviation from zero (typical of clonal population) indicates increased genetic distance between two individuals from the same seed line. Once again “*X59*” displayed the least distance between individuals indicating a possible strong selection for stable traits in the cannabinoid, fiber and grain expression pathways, and thus a good homogeneous production. For drug-type varieties, the three “*Durga Matta II CBD*” accessions, which were vegetative cuttings from the same mother plants were as expected confirmed to be identical clones.

On the other end of the spectrum, several drug-type cultivars had very large *Ia*, which may indicate mislabelling of individual plants or tremendous outcrossing, a syndrome of using F1 hybrids, which appears quite common in the industry to date.

### Association between genetic clusters and chemotypic expression

Looking through a broader lens at the 5 clusters into which the 420 samples segregate one can clearly see a strong differentiation between fiber and resin-type cannabis (Figs. 1, 2, 3, Table 1). One can infer strong selective pressure against THCA expression in K2 (CBD resin type) and K5 (Industrial hemp). Individuals in these clusters, while expressing similar chemotypes, likely underwent a bottleneck for CBDA expression, while displaying large *Ia* values, likely indicative of the polyphyletic and broad origins of the samples at hand for both the resin and fiber-type cannabis. While no chemotypic data was available for the fiber-type cultivars from K5, a subsample of 118 resin-type cultivars with chemotypic data, particularly for major cannabinoid and terpenoid expression demonstrate that (K2 CBD resin type) also consistently expressed p-cymene more so than other resin-type accessions (Fig. 4, Table 1). Among the THC expressing resin-type cluster, K4, the terpinolene dominant group also appeared to accumulate more CBGA and less CBCA than K1–3 (Fig. 4, Table 1). The latter appear to be well warranted sub-clades given the higher level of diversity observed in the aggregate K1–3 compared to each cluster individually.

## Discussion

The cannabis (2n = 2x = 20) draft genome has a haploid genomic sequence of over 876 Mb – 1000 Mb (Laverty et al. 2019; McKernan et al. 2020) and transcriptome of at least 30,000 genes (van Bakel et al. 2011; Jenkins and Orsburn 2019a, 2019b, 2019c). The genome displays a large amount of polymorphism with a single nucleotide polymorphism (SNP) present in one-in-a-hundred to one-in-fifty base pairs (McKernan et al. 2020). The phylogenetic relationship and basis for the intra-genus classification have typically recognized a broad structure with divergence between fiber-type hemp and drug/resin types cannabis (Sawler et al. 2015; Dufresnes et al. 2017). In the present study, we looked deeper into the extent and distribution of genetic diversity in modern commercial cannabis using a novel targeted genetic assay.

While often debated in the literature and confused by lore, our data support a strong historical and genome-wide division between fiber and resin-type cannabis. McPartland et al. (2018) suggested that hemp (*C. s. ruderalis*) is the ancestral group and originated in Europe about 19.7 M years ago. A combination of genetic drift and selection then likely contributed to the observed differentiation between fiber and resin cultivars (McPartland et al. 2018). The introgression of an active CBDAS into resin-type cannabis likely occurred over the past decade since the advent of medical and recreational cannabis legislation in Europe and North America. Of interest high CBD and balanced (Type II) accessions were found to cluster into the three resin groups identified here, suggesting a polyphyletic origin of high CBD resin-type cannabis. It is assumed from mapping populations that the active form of CBDAS and THCAS are at different loci on Chromosome 9, 8 cM apart in a linked tandem repeat region nestled in a complex array of transposable elements (Weiblen et al., 2015), making the characterization of this region quite complex. Further whole genome sequencing data, particularly using long reads, has enabled deeper insight into the structure of the cannabinoid cassette and demonstrates that the inactive CBDAS gene is in close linkage to the active THCAS (McKernan et al. 2020).

In addition to cannabinoid expression, another marker linked to xylan polysaccharide metabolism (SVIP14; 1–4 Beta Xylanase) was found to contribute to the separation between resin and fiber types which may play a role in fiber quality, given its putative function of breaking down the major constituent of cell walls. Such a marker may provide a possible avenue for the development of multi-purpose resin/fiber cultivars.

Integrative analyses revealed a co-expression network of genes involved in the biosynthesis of both cannabinoids and terpenoids from common precursors (Zager et al. 2019). As such, we searched for signals underlying the resin-type cannabis clusters differentiated by the dominant terpenes expression, often under the control of two dozen terpene synthase genes (TPS; Allen et al. 2019). While we did not find specific TPS linked markers, we found that a number of SNPs falling in uncharacterized regions of the current *C. sativa* genome were associated with the differentiation between terpene groups in the resin accessions sampled here. Two markers in particular showed strong differentiation between terpinolene dominant (*“sativa”*) and the other myrcene and limonene dominant accessions (*“indica”*), in particular VSSL_digi2, located in an O-glucosyltransferase Rumi analogue involved in ribosome biogenesis and SVIP16 a protein kinase possibly involved in developmental and defense-related processes.

Additionally, the chemical data available in the study supported the assertion by others (McKernan et al. 2020) that the presence/absence of a CBCAS gene in resin-type cannabis may be responsible for the “leaky” expression of THCA even in cultivars that do not contain an active copy of THCAS. As such, selection against the presence of the CBCAS may provide a possible avenue towards the development of high resin cultivars that are compliant with the current USDA / Health Canada domestic hemp production programs.

## Conclusion

We present a targeted genetic assay and algorithms related to sub-genus classification in cannabis. We demonstrate the use and repeatability of the assay to tease fiber- from resin-type cannabis as well as derive possible chemotype classes within resin-type cannabis. We demonstrate some of the utility of the assay as it related to breeding compliant cannabis and in providing a rapid means to individually identify cannabis accessions and to derive an individual fingerprint that may be used in seed-to-sale tracking and traceability endeavours. The population level data demonstrates that most resin-type varieties exhibited high heterozygosity and as such should be considered unstable at this stage. The use of our array or similar technologies will help in reducing heterozygosity and improving on the stability of trait expression in a similar manner as has been achieved in a fiber-type cultivar sampled here, with low heterozygosity and stable trait expression in large seed batches.

## Supplementary information

**Additional file 1 **: **Supplementary Figure S1.** Locus specific deviation from Hardy-Weinberg Equilibrium (HWE) for each samples seed stock. Heat map indicated *P*-value of test with pink boxes indication significant deviation from HWE. **Supplementary Figure S2.** Locus specific deviation from Hardy-Weinberg Equilibrium (HWE) for each inferred clusters. Heat map indicated P-value of test with pink boxes indication significant deviation from HWE.**Supplementary Table S1.** Seed stock specific population genetic metrics. **Supplementary Table S2.** Locus specific statistics. **Supplementary Table S3**. Table indicating the origin of each sample analysed in the study.

## Data Availability

The terpene dataset for 118 individual samples from Nevada is available at the following can be accessed here (10.6084/m9.figshare.11780103.v1). The genetic data from the 23 SNPs type in 420 individuals with no missing data can be accessed here (10.6084/m9.figshare.11778936.v1). The sequence information and cycling parameters for the 23 SNPs are available from the corresponding author upon request.

## Abbreviations

PCR: Polymerase chain reaction
SNP: Single nucleotide polymorphism
KASP: Kompetitive allele specific PCR
DAPC: Discriminant analysis of principal components
PCA: Principal component analysis
THC: Tetrahydrocannabinol
CBD: Cannabidiol

## References

[CR1] Allen KD, McKernan K, Pauli C, Roe J, Torres A, Gaudino R (2019). Genomic characterization of the complete terpene synthase gene family from *Cannabis sativa*. PLoS One.

[CR2] Altschul S, Gish W, Miller W, Myers EW, Lipman DJ (1990). Basic local alignment search tool. J Mol Biol.

[CR3] Booth JK, Bohlmann J (2019). Terpenes in *Cannabis sativa*—from plant genome to humans. Plant Sci.

[CR4] Brown AHD, Feldman MW, Nevo E (1980). Multilocus structure of natural populations of *Hordeum spontaneum*. Genetics.

[CR5] Cascini F, Farcomeni A, Migliorini D, Baldassarri L, Boschi I, Martello S, Amaducci S, Lucini L, Bernardi J (2019). Highly predictive genetic markers distinguish drug-type from fiber-type *Cannabis sativa* L. Plants.

[CR6] Clarke R, Merlin M (2013). Cannabis: evolution and ethnobotany.

[CR7] de Meijer EPM, Hammond KM (2005). The inheritance of chemical phenotype in *Cannabis sativa* L. (II): cannabigerol predominant plants. Euphytica.

[CR8] de Meijer EPM, Hammond KM, Sutton A (2009). The inheritance of chemical phenotype in *Cannabis sativa* L. (IV): cannabinoid-free plants. Euphytica.

[CR9] Dolgin E (2019). Inner workings: genomics blazes a trail to improved cannabis cultivation. PNAS.

[CR10] Dufresnes C, Jan C, Bienert F, Goudet J, Fumagalli L (2017). Broad-scale genetic diversity of *Cannabis* for forensic applications. PLoS One.

[CR11] Elzinga S, Fischedick J, Podkolinski R, Raber JC (2015). Cannabinoids and terpenes as chemotaxonomic markers in cannabis. Nat Prod Chem Res.

[CR12] Goudet J, Jombart T (2015). Package ‘hierfstat’.

[CR13] Grassa CJ, Wenger JP, Dabney C, Poplawski SG, Motley ST, Michael TP, Schwartz CJ, Weiblen GD. A complete Cannabis chromosome assembly and adaptive admixture for elevated cannabidiol (CBD) content. bioRxiv. 2018. 10.1101/458083.

[CR14] Hanuš LO, Meyer SM, Muñoz E, Taglialatela-Scafati O, Appendino G (2016). Phytocannabinoids: a unified critical inventory. Nat Prod Rep.

[CR15] Henry P (2015). Genome-wide analyses reveal clustering in Cannabis cultivars: the ancient domestication trilogy of a panacea. PeerJ PrePrints.

[CR16] Henry P (2017). Cannabis chemovar classification: terpenes hyper-classes and targeted genetic markers for accurate discrimination of flavours and effects. PeerJ Preprints.

[CR17] Henry P, Hilyard A, Johnson S, Orser C (2018). Predicting chemovar cluster and variety verification in vegetative cannabis accessions using targeted single nucleotide polymorphisms. PeerJ Preprints.

[CR18] Hilyard A, Lewin S, Johnson S, Henry P, Orser C (2019). Application of a simple genetic assay to discriminate hemp from drug-type cannabis. Cannabis Sci Technol.

[CR19] Jenkins C, Orsburn B. The cannabis multi-omics draft map project. bioRxiv. 2019a:753400 10.1101/753400.

[CR20] Jenkins C, Orsburn B. The first publicly available annotated genome for cannabis plants. bioRxiv. 2019b:786186 10.1101/786186.

[CR21] Jenkins C, Orsburn B. Constructing a draft map of the cannabis proteome. bioRxiv. 2019c:577635 10.1101/577635.

[CR22] Jombart T (2008). Adegenet: a R package for the multivariate analysis of genetic markers. Bioinformatics.

[CR23] Kamvar ZN, Tabima JF, Grünwald NJ (2014). Poppr: an R package for genetic analysis of populations with clonal, partiallyclonal, and/or sexual reproduction. PeerJ.

[CR24] Laverty KU, Stout JM, Sullivan MJ, Shah H, Gill N, Holbrook L, Page J, van Bakel H (2019). A physical and genetic map of *Cannabis sativa* identifies extensive rearrangements at the *THC/CBD acid synthase* loci. Genome Res.

[CR25] Lewis MA, Russo EB, Smith KM (2018). Pharmacological foundations of Cannabis chemovars. Planta Med.

[CR26] Lynch RC, Vergara D, Tittes S, White K, Schwartz CJ, Gibbs MJ, Ruthenburg TC, deCesare K, Land DP, Kane NC (2016). Genomic and chemical diversity in *Cannabis*. Crit Rev Plant Sci.

[CR27] McKernan KJ, Helbert Y, Kane LT, Ebling H, Zhang L, Liu B, Eaton Z, McLaughlin S, Kingan S, Baybayan P, Concepcion G, Jordan M, Riva A, Barbazuk W, Harkins T. Sequence and annotation of 42 cannabis genomes reveals extensive copy number variation in cannabinoid synthesis and pathogen resistance genes. bioRxiv. 2020:2020.01.03.894428 10.1101/2020.01.03.894428.

[CR28] McPartland J, Guy GW, Hegman W (2018). *Cannabis* is indigenous to Europe and cultivation began during the copper or bronze age: a probabilistic synthesis of fossil pollen studies. Veg Hist Archaeobotany.

[CR29] McPartland JM (2018). *Cannabis* systematics at the levels of family, genus, and species. Cannabis Cannabinoid Res.

[CR30] Onofri C, Mandolino G, Chandra S, Lata H, ElSohly MA (2017). Genomics and molecular markers in *Cannabis sativa* L. *Cannabis sativa* L -botany and biotechnology.

[CR31] Orser C, Henry P (2019). Making sense of cannabis strains through chemometrics. Cannabis Sci Technol.

[CR32] Orser C, Johnson S, Speck M, Hilyard A, Afia I (2018). Terpenoid chemoprofiles distinguish drug-type *Cannabis sativa* L. cultivars in Nevada. Nat Prod Chem Res.

[CR33] Paradis E (2010). pegas: an R package for population genetics with an integrated-modular approach. Bioinformatics.

[CR34] Paradis E, Schliep K (2018). ape 5.0: an environment for modern phylogenetics and evolutionary analyses in R. Bioinformatics.

[CR35] R Core Team (2018). R: A language and environment for statistical computing.

[CR36] Rahn B, Pearson BJ, Trigiano RN, Gray DJ (2016). The derivation of modern Cannabis varieties. Crit Rev Plant Sci.

[CR37] Sawler J, Stout JM, Gardner KM, Hudson D, Vidmar J, Butler L, Page JE, Myles S (2015). The genetic structure of marijuana and hemp. PLoS One.

[CR38] Schwabe AL, McGlaughlin ME (2019). Genetic tools weed out misconceptions of strain reliability in *Cannabis sativa:* implications for a budding industry. J Cannabis Res.

[CR39] Toth JA, Stack GM, Cala AR, Carson CH, Wilk RL, Crawford JL, Viands DR, Philippe G, Smart CD, Rose JKC, Smart LB (2020). Development and validation of genetic markers for sex and cannabinoid chemotype in *Cannabis sativa* L. GCB Bioenergy.

[CR40] van Bakel H, Stout JM, Cote AG, Tallon CM, Sharpe AG, Hughes TR (2011). The draft genome and transcriptome of *Cannabis sativa*. Genome Biol.

[CR41] Weiblen GD, Wenger JP, Craft KJ, ElSohly MA, Mehmedic Z, Treiber EL, Marks MD (2015). Gene duplication and divergence affecting drug content in Cannabis sativa. The New Phytologist.

[CR42] Zager JJ, Lange I, Srividya N, Smith A, Lange BM (2019). Gene networks underlying cannabinoid and terpenoid accumulation in cannabis. Plant Physiol.

