# Supplementary material for: A single nucleotide polymorphism assay sheds light on the extent and distribution of genetic diversity, population structure and functional basis of key traits in cultivated north American cannabis
Source: J Cannabis Res. 2020 Sep 11;2:26. doi: 10.1186/s42238-020-00036-y (PMC7819309; doi:10.1186/s42238-020-00036-y)
Supplement: Supplementary file 1 — Additional file 1 : Supplementary Figure S1. Locus specific deviation from Hardy-Weinberg Equilibrium (HWE) for each samples seed stock. Heat map indicated P-value of test with pink boxes indication significant deviation from HWE. Supplementary Figure S2. Locus specific deviation from Hardy-Weinberg Equilibrium (HWE) for each inferred clusters. Heat map indicated P-value of test with pink boxes indication significant deviation from HWE.Supplementary Table S1. Seed stock specific population genetic metrics. Supplementary Table S2. Locus specific statistics. Supplementary Table S3. Table indicating the origin of each sample analysed in the study. [file 42238_2020_36_MOESM1_ESM.pdf]

Supplementary Material Figure S1:

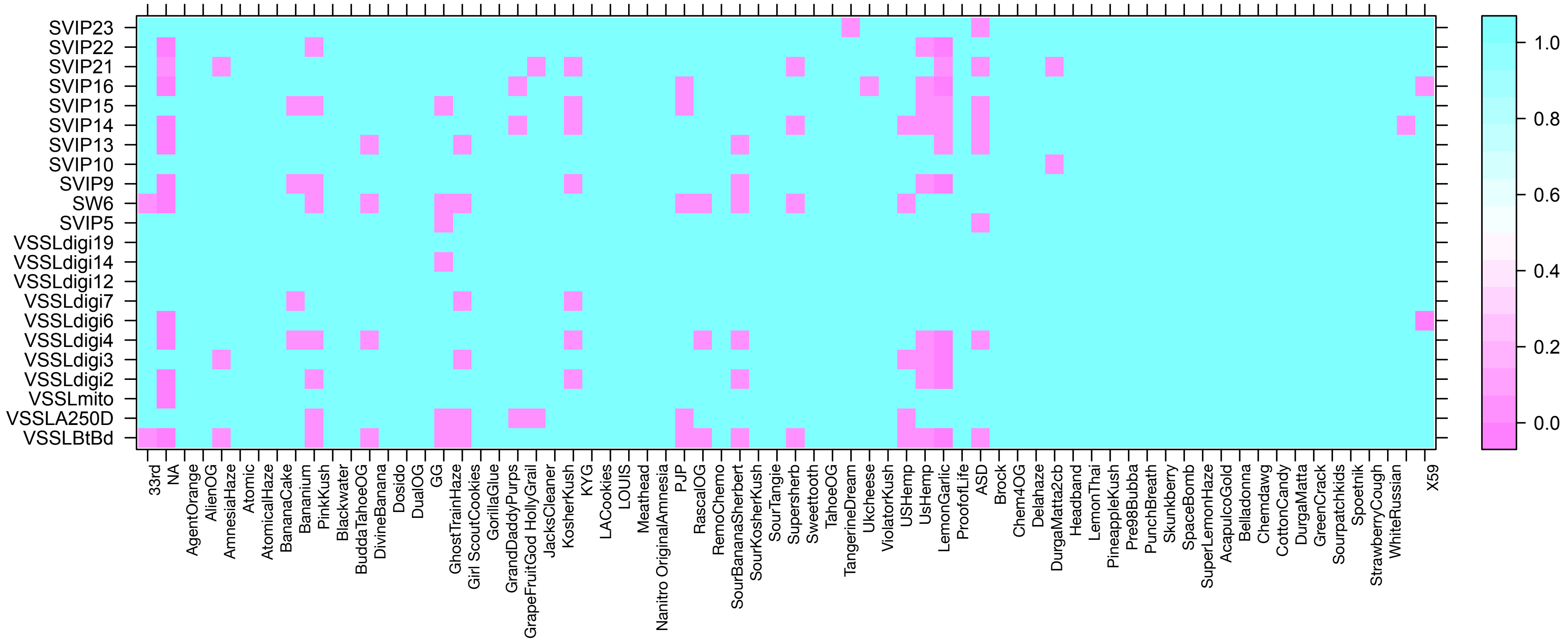

Supplementary material Figure S2:

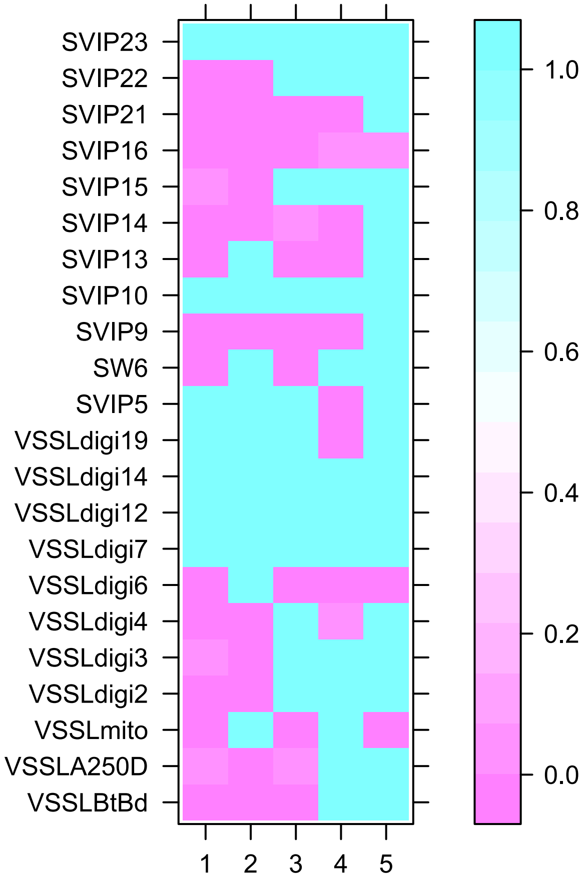

Supplementary Material Table S1

**Pop**	**N**	**MLG**	**Hexp**	**Ia**
**33rd**	5	5	**0.190909091**	1.580487805
**AmnesiaHaze**	4	4	0.381493506	0.725925926
**ASD**	4	3	**0.188311688**	2.217391304
**AtomicalHaze**	3	3	0.275757576	1.818181818
**BananaCake**	3	3	0.284848485	0.625
**Bananium**	5	5	0.246464646	0.475
**Blackwater**	3	3	0.281818182	2
**BuddaTahoeOG**	3	3	0.327272727	-0.2
**Chem4OG**	3	3	**0.163636364**	2.5
**CottonCandy**	3	3	**0.133333333**	**0.75**
**Delahaze**	5	4	0.27979798	3.058020478
**DivineBanana**	4	4	0.282467532	1.402298851
**Dosido**	3	3	0.321212121	1.6
**DualOG**	6	6	0.289944904	0.8125
**DurgaMatta**	3	2	**0.2**	8
**DurgaMatta2cb**	3	1	**0.190909091**	**0**
**GhostTrainHaze**	4	4	0.355519481	1.663366337
**Girl coutCookies**	7	7	0.308191808	1.30044843

Pop	N	MLG	Hexp	Ia
**GorillaGlue**	3	3	0.266666667	3.454545455
**GrapeFruitGod**	4	4	0.258116883	1.454545455
**HollyGrail**	4	4	0.285714286	-0.295652174
**JacksCleaner**	3	3	0.275757576	0.625
**KosherKush**	4	4	0.331168831	0.225806452
**KYG**	3	3	0.363636364	-0.8
**LACookies**	5	5	0.294949495	-0.032171582
**LOUIS**	3	3	0.272727273	8.25
**Meathead**	2	2	0.310606061	NA
**Nanitro**	3	3	0.260606061	6.3
**OriginalAmnesia**	5	4	0.214141414	1.197452229
**PineappleKush**	3	3	0.3	0.5
**PinkKush**	7	3	**0.18981019**	1.5
**PJP**	4	4	0.225649351	0.03030303
**ProofofLife**	4	2	0.266233766	3
**PunchBreath**	3	2	**0.136363636**	2
**RemoChemo**	4	4	0.251623377	-0.677419355
**SourTangie**	7	7	0.283716284	0.725943971
**SpaceBomb**	3	3	0.233333333	1.8

Pop	N	MLG	Hexp	Ia
**StrawberryCough**	3	3	**0.124242424**	**0.75**
**Sweettooth**	3	3	0.351515152	1.153846154
**TahoeOG**	5	5	0.211111111	1.482758621
**TangerineDream**	9	9	0.322638146	0.840565086
**UsHemp**	20	11	0.279545455	0.994321219
**USHemp**	7	6	0.293706294	1.635103926
**ViolatorKush**	7	7	0.303696304	1.38769671
**WhiteRussian**	7	7	**0.184315684**	**0.327198364**
**X59**	14	14	**0.093073593**	**0.167832168**
**Total / average**	420	361	0.330011764	0.570375586

Supplementary Material Table S2

SNP	allele	1-D	Hexp	Evenness
**VSSLBtBd**	2	0.486111111	0.486690505	0.97290917
**VSSLA250D**	2	0.318568594	0.318948294	0.722946759
**VSSLmito**	2	0.073287982	0.073375333	0.450170654
**VSSLdigi2**	2	0.404036281	0.40451785	0.836002253
**VSSLdigi3**	2	0.497276077	0.497868778	0.994579203
**VSSLdigi4**	2	0.427437642	0.427947103	0.871561439
**VSSLdigi6**	2	0.075484694	0.075574664	0.453144701
**VSSLdigi7**	2	0.25997449	0.260284352	0.656034447
**VSSLdigi12**	2	0.04875	0.048808105	0.413244143
**VSSLdigi14**	2	0.136723356	0.136886316	0.52560179
**VSSLdigi19**	2	0.025847506	0.025878313	0.367110514
**SVIP5**	2	0.499770408	0.500366082	0.99954101
**SW6**	2	0.49744898	0.498041887	0.994921692
**SVIP9**	2	0.470510204	0.471071003	0.94401974
**SVIP10**	2	0.128560091	0.128713321	0.5166806
**SVIP13**	2	0.485306122	0.485884556	0.971379846
**SVIP14**	2	0.355226757	0.35565015	0.768669616
**SVIP15**	2	0.499657029	0.500252568	0.99931449

SNP	allele	1-D	Hexp	Evenness
**SVIP16**	2	0.447573696	0.448107157	0.90420323
**SVIP21**	2	0.409180839	0.40966854	0.843617072
**SVIP22**	2	0.317131519	0.317509507	0.721223835
**SVIP23**	2	0.387752268	0.388214428	0.812593468
**mean**	2	0.329618893	0.330011764	0.760884985

allele = Number of observed alleles
Hexp - Nei's gene diversity (expected heterozygosity)
Evenness - Index of Association for each population factor

Supplementary Material Table S3

K	Producer	Origin	Pop	Ind
3	Emerald Flower	BC	33rd	33rd1
1	Emerald Flower	BC	33rd	33rd10
3	Emerald Flower	BC	33rd	33rd6
3	Emerald Flower	BC	33rd	33rd7
3	Emerald Flower	BC	33rd	33rd8
1	Acres	NV	NA	5thDimension
1	Nature's Chemistry	NV	NA	702OG
3	GLP	NV	NA	9LBHammer
4	VSSL	BC	AcapulcoGold	aca2
4	VSSL	BC	AcapulcoGold	AcapulcoGold
2	Happy Campers	NV	NA	Adam
1	Flowr	BC	AgentOrange	Agentorange1
1	Nature's Chemistry	NV	NA	AirForceOne
3	Emerald Flower	BC	NA	AK59
2	Yield Farming	NV	USHemp	Alamo
2	Yield Farming	NV	USHemp	Alamo1
4	Flowr	BC	AlienOG	AlienOG10
1	Flowr	BC	AlienOG	AlienOG9

K	Producer	Origin	Pop	Ind
4	Flowr	BC	ASD	AmherstSourDiesel
1	Flowr	BC	AmnesiaHaze	Amnesia
1	Flowr	BC	AmnesiaHaze	AmnesiaHaze_a
4	Flowr	BC	AmnesiaHaze	AmnesiaHaze12
3	Flowr	BC	AmnesiaHaze	AmnesiaHaze13
4	Flowr	BC	ASD	ASD1_a
4	Flowr	BC	ASD	ASD2_a
3	Flowr	BC	ASD	ASD3_a
1	Flowr	BC	Atomic	Atomic15
1	Flowr	BC	Atomic	Atomic17
4	Flowr	BC	AtomicalHaze	AtomicalHaze
1	Flowr	BC	AtomicalHaze	AtomicalHaze_a
4	Flowr	BC	AtomicalHaze	AtomicalHaze_b
1	Okanagan Gold	BC	NA	AyawaskaPurple
1	Emerald Flower	BC	BananaCake	BananaCake1
1	Emerald Flower	BC	BananaCake	BananaCake4
1	Emerald Flower	BC	BananaCake	BananaCake5
1	Nature's Chemistry	NV	NA	BananaPunch
1	CCLV	NV	NA	BananaTreez

K	Producer	Origin	Pop	Ind
4	Emerald Flower	BC	Bananium	Bananium10
4	Emerald Flower	BC	Bananium	Bananium11
1	Emerald Flower	BC	Bananium	Bananium2
1	Emerald Flower	BC	Bananium	Bananium4
1	Emerald Flower	BC	Bananium	Bananium7
4	Flowr	BC	Belladonna	Belladonna_a
4	Flowr	BC	Belladonna	Belladonna_b
2	Emerald Flower	BC	NA	Berry Blossum
1	Okanagan Gold	BC	PinkKush	BestShitEver
1	Purefarma	BC	PinkKush	BillyCrystal
1	Franklin Bioscience	NV	NA	BlackberryCream
4	Flowr	BC	Blackwater	Blackwater18
1	Flowr	BC	Blackwater	Blackwater19
1	Flowr	BC	Blackwater	Blackwater20
3	Green and Gold	NV	NA	BlackwaterOG
3	Green and Gold	NV	NA	BloodDrive
3	Emerald Flower	BC	NA	Blue
1	Green and Gold	NV	NA	BlueAlien
1	CCLV	NV	NA	Blueberry

K	Producer	Origin	Pop	Ind
4	Emerald Flower	NV	NA	Blueberry1
3	Nature's Chemistry	NV	NA	BlueberryCookies
1	Emerald Flower	BC	NA	BlueberryKush
1	Green and Gold	NV	NA	BlueCheese
3	Green and Gold	NV	NA	BlueDream
3	Green and Gold	NV	NA	BlueHashPlant
3	Green and Gold	NV	NA	BlueHashPlant1
1	Nature's Chemistry	NV	NA	BlueZkittlez
2	Western State Hemp	NV	USHemp	Boax
3	Flowr	BC	Brock	Brock_a
3	Flowr	BC	Brock	BrockxSA_b
1	Acres	NV	NA	BuckeyePurple
3	Flowr	BC	BuddaTahoeOG	BuddaTahoeOG_a
4	Flowr	BC	BuddaTahoeOG	BuddhaTahoeOG21
1	Flowr	BC	BuddaTahoeOG	BuddhaTahoeOG22
3	Emerald Flower	BC	NA	CB7
4	Flowr	BC	NA	CBDzen_a
3	Flowr	BC	NA	CBDzen_b
1	Terra	BC	NA	CBOG

K	Producer	Origin	Pop	Ind
1	Thompson Farm One	NV	NA	CementShoes
1	Acres	NV	NA	Chem 91
1	Franklin Bioscience	NV	NA	Chem4
3	Flowr	BC	Chem4OG	Chem4OG23
3	Flowr	BC	Chem4OG	Chem4OG24
4	Flowr	BC	Chem4OG	Chem4OG25
4	Emerald Flower	BC	Chemdawg	chemdawg1
4	Franklin Bioscience	NV	NA	CherryDiesel
1	Emerald Flower	BC	NA	CherryPie
2	Hemp Inc.	NV	UsHemp	CherryWine_las
2	Flowr	BC	NA	Cindy99_a
1	Foreman	BC	NA	CitrusFarmer
1	Acres	NV	NA	ColoradoSunset
4	Flowr	BC	CottonCandy	CottonCandy30
4	Flowr	BC	CottonCandy	CottonCandy31
4	Flowr	BC	CottonCandy	CottonCandy33
1	Okanagan Gold	BC	NA	CrownRoyal
1	Greenway	NV	NA	CuriousGeorge7
1	Emerald Flower	BC	NA	d_CR

K	Producer	Origin	Pop	Ind
1	Emerald Flower	BC	NA	d_PC
3	Emerald Flower	BC	NA	d_RKS
1	Emerald Flower	BC	NA	d_SC1
3	Emerald Flower	BC	NA	d2
3	Emerald Flower	BC	NA	d5
4	Emerald Flower	BC	NA	d6
1	Emerald Flower	BC	NA	d7
4	Emerald Flower	BC	NA	d8
3	Emerald Flower	BC	NA	Dance
3	Okanagan Gold	BC	NA	DanceHall
5	In Planta	AB	NA	darryl
3	Flowr	BC	Delahaze	Delahaze_a
3	Flowr	BC	Delahaze	Delahaze_b
4	Flowr	BC	Delahaze	Delahaze35
4	Flowr	BC	Delahaze	Delahaze37
4	Flowr	BC	Delahaze	Delahaze38
1	Emerald Flower	BC	DivineBanana	DivineBanana3
1	Emerald Flower	BC	DivineBanana	DivineBanana5
1	Emerald Flower	BC	DivineBanana	DivineBanana6

K	Producer	Origin	Pop	Ind
1	Emerald Flower	BC	DivineBanana	DivineBanana7
3	Green and Gold	NV	NA	DJsGold
1	Terra	BC	Dosido	Dosidos_a
1	Terra	BC	Dosido	Dosidos2
1	Matrix	NV	Dosido	Dosidoslas
4	Emerald Flower	BC	DualOG	DualOG10
1	Emerald Flower	BC	DualOG	DualOG2
2	Emerald Flower	BC	DualOG	DualOG3
4	Emerald Flower	BC	DualOG	DualOG4
4	Emerald Flower	BC	DualOG	DualOG8
4	Emerald Flower	BC	DualOG	DualOG9
4	Flowr	BC	DurgaMatta	DurgaMatta_a
4	Flowr	BC	DurgaMatta	DurgaMatta_b
4	Flowr	BC	DurgaMatta	DurgaMatta_c
3	Flowr	BC	DurgaMatta2cb	DurgaMatta2cbd_a
3	Flowr	BC	DurgaMatta2cb	DurgaMatta2cbd_b
3	Flowr	BC	DurgaMatta2cb	DurgaMatta2cbd_c
1	Emerald Flower	BC	NA	DutchTreat
3	Okanagan Gold	BC	NA	Dweed

K	Producer	Origin	Pop	Ind
3	Island Genetics	BC	NA	Egypt
4	Terra	BC	NA	ElmersGlue
1	Greenway	NV	NA	French Bread
3	Good Uncle	BC	NA	fruitychronic
1	Emerald Flower	BC	NA	Garlic
3	Emerald Flower	BC	NA	GDP
1	Terra	BC	NA	GelatoBreath
1	Emerald Flower	BC	NA	GF
1	Emerald Flower	BC	GG	GG4
3	Emerald Flower	BC	GG	GG5
4	Purefarma	BC	NA	GGC
1	Flowr	BC	GhostTrainHaze	GhostTrainHaze_a
3	Flowr	BC	GhostTrainHaze	GhostTrainHaze_d
3	Flowr	BC	GhostTrainHaze	GhostTrainHaze_f
3	Nature's Chemistry	NV	GhostTrainHaze	GhostTrainHaze_las
1	Flowr	BC	Girl coutCookies	Girl coutCookies1
1	Flowr	BC	Girl coutCookies	Girlscootcookies2
3	Flowr	BC	Girl coutCookies	GirlScoutCookies40
3	Flowr	BC	Girl coutCookies	GirlScoutCookies41

K	Producer	Origin	Pop	Ind
3	Flowr	BC	Girl coutCookies	GirlScoutCookies42
3	Flowr	BC	Girl coutCookies	GirlScoutCookies43
3	Flowr	BC	Girl coutCookies	GirlScoutCookies45
1	GLP	NV	NA	GlueOnFire
3	Emerald Flower	BC	NA	GodBud
2	Green and Gold	NV	NA	GoldenLemons
1	Franklin Bioscience	NV	GorillaGlue	GorillaGlue
4	Flowr	BC	GorillaGlue	GorillaGlue_a
3	Flowr	BC	GorillaGlue	GorillaGlue446
1	Flowr	BC	GrandDaddyPurps	GrandDaddyPurps49
1	Flowr	BC	GrandDaddyPurps	GrandDaddyPurps50
3	Good Uncle	BC	NA	Grapefruit
4	Flowr	BC	GrapeFruitGod	GrapeFruitGod_a
4	Flowr	BC	GrapeFruitGod	GrapeFruitGod_b
4	Flowr	BC	GrapeFruitGod	GrapefruitGod53
1	Flowr	BC	GrapeFruitGod	GrapefruitGod56
1	GLP	NV	NA	GratefulBreath
4	Flowr	BC	GreenCrack	GreenCrack_a
2	Western State Hemp	NV	UsHemp	HammermilledBiomassBag11

K	Producer	Origin	Pop	Ind
2	Western State Hemp	NV	UsHemp	HammermilledBiomassBag7
3	Flowr	BC	Headband	Headband1
4	Flowr	BC	Headband	Headband63
1	Polaris MMJ	NV	NA	HeadCheese
1	Greenway	NV	NA	HellsOG
2	Harris Farms	NV	UsHemp	Hemp1
2	Harris Farms	NV	UsHemp	Hemp10
2	Harris Farms	NV	UsHemp	Hemp11
2	Harris Farms	NV	UsHemp	Hemp12
2	Harris Farms	NV	UsHemp	Hemp13
2	Harris Farms	NV	UsHemp	Hemp14
2	Harris Farms	NV	UsHemp	Hemp15
2	Harris Farms	NV	UsHemp	Hemp16
2	Harris Farms	NV	UsHemp	Hemp2
2	Harris Farms	NV	UsHemp	Hemp3
2	Leadfceuticals	NV	UsHemp	Hemp4
2	Leafceuticals	NV	UsHemp	Hemp5
2	Happy Campers	NV	UsHemp	Hemp6
2	Happy Campers	NV	UsHemp	Hemp7

K	Producer	Origin	Pop	Ind
2	Calineva Farms	NV	UsHemp	Hemp9
2	Calineva Farms	NV	UsHemp	HempBin
2	Calineva Farms	NV	UsHemp	HempBucket
1	Emerald Flower	BC	NA	High
4	Island Genetics	BC	NA	Himalaya
4	Island Genetics	BC	NA	HinduKush
4	Flowr	BC	HollyGrail	HolyGrail_a
4	Flowr	BC	HollyGrail	HolyGrail_b
1	Green and Gold	NV	HollyGrail	HolyGrailKush_las
3	Flowr	BC	HollyGrail	HolyGrailKush64
1	Flowr	BC	NA	IceCream
4	CCLV	NV	NA	J1
4	Franklin Bioscience	NV	NA	Jack Herer
3	Flowr	BC	JacksCleaner	JacksCleaner65
1	Flowr	BC	JacksCleaner	JacksCleaner66
1	Flowr	BC	JacksCleaner	JacksCleaner67
1	Greenway	NV	NA	JamaicanTenspeed
1	Thompson Farm One	NV	NA	JetFuel
1	Green and Gold	NV	NA	JohnnyChimpo

K	Producer	Origin	Pop	Ind
1	CCLV	NV	NA	KeyLimePie
1	Flowr	BC	KosherKush	KosherKush_b
1	Flowr	BC	KosherKush	KosherKush70
4	Flowr	BC	KosherKush	KosherKush71
1	Flowr	BC	KosherKush	KosherKush72
3	Green and Gold	NV	NA	Kushage
5	Digipath	NV	KYG	KYG-G
2	Digipath	NV	KYG	KYG-M
1	Digipath	NV	KYG	KYG-T
4	Flowr	BC	LACookies	LACookies73
3	Flowr	BC	LACookies	LACookies74
1	Flowr	BC	LACookies	LACookies75
2	Flowr	BC	LACookies	LACookies76
2	Flowr	BC	LACookies	LACookies77
1	Emerald Flower	BC	NA	LAlights
3	Green and Gold	NV	NA	LaPlatina
3	Nature's Chemistry	NV	NA	LemonBananaSherbert
2	Emerald Flower	BC	LemonGarlic	LemonGarlic
3	Purefarma	BC	LemonThai	LemonThai

K	Producer	Origin	Pop	Ind
4	Flowr	BC	LemonThai	LemonThai1
1	Polaris MMJ	NV	NA	LemonWalkerOG
3	CCLV	NV	NA	LOrange
1	Thompson Farm One	NV	LOUIS	LouieVIII
1	Greenway	NV	LOUIS	LouieXIIIOG
4	Flowr	BC	LOUIS	LouisXIII78
3	GLP	NV	NA	LoveTriangle
1	Emerald Flower	BC	NA	lsd
2	Happy Campers	NV	NA	LV1Ground
2	Happy Campers	NV	NA	LV1PregroundA
4	Emerald Flower	BC	NA	M1
1	GLP	NV	NA	MAC
1	Greenway	NV	NA	MangoTango
1	Emerald Flower	BC	Meathead	Meathead8
1	Emerald Flower	BC	Meathead	Meathead9
3	Emerald Flower	BC	NA	Medic
1	Green Harvest	NV	NA	Megaslex
3	Emerald Flower	BC	NA	MemoryLoss
1	Terra	BC	NA	MendoBreath

K	Producer	Origin	Pop	Ind
2	GLP	NV	NA	MissX
1	Greenway	NV	NA	Motorbreath
1	Emerald Flower	BC	Nanitro	Nanitro11
3	Emerald Flower	BC	Nanitro	NAnitro12
3	Emerald Flower	BC	Nanitro	Nanitro8
1	Matrix	NV	NA	NepaleseKush
2	Flowr	BC	NA	NinaXstrw_a
3	Emerald Flower	BC	NA	NN
3	Green and Gold	NV	NA	NorthernLights
3	Acres	NV	NA	NorthernRussian
1	Green and Gold	NV	NA	OGKosher
3	Flowr	BC	NA	OGKush80
3	Terra	BC	NA	OrangeApricot
1	GLP	NV	NA	OrangeCookies
1	Green Harvest	NV	NA	OrangeCreamsicle
1	CCLV	NV	NA	OrangeZeta
3	Flowr	BC	OriginalAmnesia	OriginalAmnesia_c
3	Flowr	BC	OriginalAmnesia	OriginalAmnesia_d
3	Flowr	BC	OriginalAmnesia	OriginalAmnesia1

K	Producer	Origin	Pop	Ind
3	Flowr	BC	OriginalAmnesia	OriginalAmnesia83
1	Flowr	BC	OriginalAmnesia	OriginalAmnesia84
1	Island Genetics	BC	NA	Panama1
1	Island Genetics	BC	NA	Panama2
1	Emerald Flower	BC	NA	PC
3	Flowr	BC	PineappleKush	PineappleKush_a
3	Flowr	BC	PineappleKush	PineappleKush_b
3	Flowr	BC	PineappleKush	PineappleKush_c
1	Purefarma	BC	PinkKush	Pink
1	Flowr	BC	PinkKush	PinkKush_a
1	Flowr	BC	PinkKush	PinkKush_b
1	Flowr	BC	PinkKush	PinkKush_c
1	Flowr	BC	PinkKush	PinkKush_d
1	Emerald Flower	BC	PJP	PJP4
1	Emerald Flower	BC	PJP	PJP5
3	Emerald Flower	BC	PJP	PJP7
1	Emerald Flower	BC	PJP	PJP8
1	Green and Gold	NV	NA	PlatinumCookies
3	Flowr	BC	NA	POGxSA_b

K	Producer	Origin	Pop	Ind
3	Flowr	BC	Pre98Bubba	Pre98Bubba87
3	Flower One	NV	ProofofLife	ProofofLife1a
3	Flower One	NV	ProofofLife	ProofofLife1b
2	Flower One	NV	ProofofLife	ProofofLife2a
2	Flower One	NV	ProofofLife	ProofofLife2b
3	Terra	BC	PunchBreath	PunchBreath1
3	Terra	BC	PunchBreath	PunchBreath2
3	Terra	BC	PunchBreath	PunchBreathMale
3	Green and Gold	NV	NA	PureKush
1	Emerald Flower	BC	NA	PurpleChunk
2	VSSL	BC	NA	PurpleDieselCBDmale
4	Emerald Flower	BC	NA	PurpleGod
1	Acres	NV	NA	PurpleHoe
3	Green and Gold	NV	NA	PurpleMonkey
4	CCLV	NV	NA	PurplePunch
3	GLP	NV	NA	PurpleWookie
1	Flowr	BC	RascalOG	RascalsOG89
1	Flowr	BC	RemoChemo	RemoChemo91
1	Flowr	BC	RemoChemo	RemoChemo92

K	Producer	Origin	Pop	Ind
1	Flowr	BC	RemoChemo	RemoChemo93
1	Flowr	BC	RemoChemo	RemoChemo94
3	Emerald Flower	BC	NA	RKS
1	Greenway	NV	NA	RocketFuel
5	VSSL	BC	NA	S2
3	Terra	BC	NA	ScoutMaster
1	Flowr	BC	NA	SensiStar
4	Flowr	BC	NA	SFVOG_a
1	Flowr	BC	NA	SFVOG_b
2	Happy Campers	NV	USHemp	Shore
2	Happy Campers	NV	USHemp	Shore1
1	Purefarma	BC	NA	SillyWabbit
3	Greenway	NV	Skunkberry	Skunkberry
3	Greenway	NV	Skunkberry	Skunkberry1
1	Franklin Bioscience	NV	NA	SlimeDawgMillaNaire
1	Green and Gold	NV	SourBananaSherbert	SourBananaSherbert
1	Green and Gold	NV	SourBananaSherbert	SourBananaSherbert1
1	Okanagan Gold	BC	NA	SourBlueberry
3	Flowr	BC	SourKosherKush	SourKosherKush_a

K	Producer	Origin	Pop	Ind
1	Flowr	BC	SourKosherKush	SourKosherKush96
4	Emerald Flower	BC	Sourpatchkids	Sourpatchkids
3	Flowr	BC	SourTangie	SourTangie_a
3	Flowr	BC	SourTangie	SourTangie_d
3	Flowr	BC	SourTangie	SourTangie100
4	Flowr	BC	SourTangie	SourTangie101
1	Flowr	BC	SourTangie	SourTangie102
3	Flowr	BC	SourTangie	SourTangie103
1	Flowr	BC	SourTangie	SourTangie104
3	Flowr	BC	SpaceBomb	SpaceBomb105
3	Flowr	BC	SpaceBomb	SpaceBomb106
4	Flowr	BC	SpaceBomb	SpaceBomb107
3	Flowr	BC	NA	SpaceDawg108
4	Franklin Bioscience	NV	NA	SpaceQueen
3	Emerald Flower	BC	NA	Special
4	Flowr	BC	Spoetnik	Spoetnik
1	CCLV	NV	NA	StardustOG
4	Flowr	BC	StrawberryCough	StrawberryCough109
4	Flowr	BC	StrawberryCough	StrawberryCough110

K	Producer	Origin	Pop	Ind
4	Flowr	BC	StrawberryCough	StrawberryCough111
1	CCLV	NV	NA	StrawberryLemonade
1	Matrix	NV	NA	SugarTits
4	Matrix	NV	NA	SugarTitsFrozen
3	Flowr	BC	SuperLemonHaze	SuperLemonHaze_a
4	Flowr	BC	SuperLemonHaze	SuperLemonHaze112
1	Emerald Flower	BC	Supersherb	SuperSherb
2	Matrix	NV	NA	SuperSourDiesel
1	Matrix	NV	NA	SweetPink
1	Flowr	BC	Sweettooth	Sweettooth_a
1	Flowr	BC	Sweettooth	Sweettooth_b
3	Flowr	BC	Sweettooth	Sweettooth_d
1	Emerald Flower	BC	NA	Tahoe
4	Flowr	BC	TahoeOG	TahoeOG115
4	Flowr	BC	TahoeOG	TahoeOG116
1	Flowr	BC	TahoeOG	TahoeOG117
4	Flowr	BC	TahoeOG	TahoeOG118
3	Flowr	BC	TahoeOG	TahoeOG119
3	Flowr	BC	TangerineDream	TangerineDream_a

K	Producer	Origin	Pop	Ind
3	Flowr	BC	TangerineDream	TangerineDream_b
4	Flowr	BC	TangerineDream	TangerineDream_c
4	Flowr	BC	TangerineDream	TangerineDream_d
3	Flowr	BC	TangerineDream	TangerineDream120
4	Flowr	BC	TangerineDream	TangerineDream121
1	Flowr	BC	TangerineDream	TangerineDream122
3	Flowr	BC	TangerineDream	TangerineDream123
4	Flowr	BC	TangerineDream	TangerineDream124
4	Purefarma	BC	NA	Thailights
3	Purefarma	BC	NA	ThaiPicnick
1	Thompson Farm One	NV	NA	ThinMint
4	Franklin Bioscience	NV	NA	TKStarDawgHaze
3	Emerald Flower	BC	NA	Tonic
1	Emerald Flower	BC	NA	Trainwreck
3	Emerald Flower	BC	NA	Treat
2	Green and Gold	NV	NA	TripleGorillaCookies
1	CCLV	NV	NA	Tropicanna
3	Purefarma	BC	NA	TropicThunder
1	Polaris MMJ	NV	NA	TrueOG

K	Producer	Origin	Pop	Ind
1	Greenway	NV	Ukcheese	UKCheese2
3	VSSL	BC	NA	UnicornCBD
1	Matrix	NV	NA	UV
1	Matrix	NV	NA	UV1
1	GLP	NV	NA	VCM
3	Flowr	BC	ViolatorKush	ViolatorKush_a
1	Flowr	BC	ViolatorKush	ViolatorKush1
3	Flowr	BC	ViolatorKush	ViolatorKush125
3	Flowr	BC	ViolatorKush	ViolatorKush126
3	Flowr	BC	ViolatorKush	ViolatorKush127
1	Flowr	BC	ViolatorKush	ViolatorKush128
1	Flowr	BC	ViolatorKush	ViolatorKush129
3	Good Uncle	BC	NA	w3
1	Good Uncle	BC	NA	w4
3	Good Uncle	BC	NA	w5
2	Western State Hemp	NV	USHemp	WesternStatesHemp
2	Western State Hemp	NV	USHemp	WesternStatesHempBiomass
4	Flowr	BC	WhiteRussian	WhiteRussian_c
4	Flowr	BC	WhiteRussian	WhiteRussian_d

K	Producer	Origin	Pop	Ind
4	Flowr	BC	WhiteRussian	WhiteRussian130
4	Flowr	BC	WhiteRussian	WhiteRussian131
4	Flowr	BC	WhiteRussian	WhiteRussian132
4	Flowr	BC	WhiteRussian	WhiteRussian133
4	Flowr	BC	WhiteRussian	WhiteRussian134
1	Green Harvest	NV	NA	Wildberrykush
3	Good Uncle	BC	NA	WLKush
5	Flying Creek	SK	X59	X59_1
5	Flying Creek	SK	X59	X59_10
5	Flying Creek	SK	X59	X59_11
5	Flying Creek	SK	X59	X59_12
5	Flying Creek	SK	X59	X59_2
5	Flying Creek	SK	X59	X59_3
5	Flying Creek	SK	X59	X59_4
5	Flying Creek	SK	X59	X59_5
5	Flying Creek	SK	X59	X59_6
5	Flying Creek	SK	X59	X59_7
5	Flying Creek	SK	X59	X59_8
5	Flying Creek	SK	X59	X59_9

K	Producer	Origin	Pop	Ind
5	Purefarma	BC	X59	X59ind
5	Purefarma	BC	X59	X59sat
4	GLP	NV	NA	ZellysGift

Cultivation partners: Emerald Flower Farm, Terra Labs, Foreman Farms, Flying Creek Trading, Purefarma Solutions, Good Uncle Green Eyes, Matrix NV, GLP, Greenway, CCLV, Green and Gold, Nature's Chemistry, Western State Hemp, Harris Farms, Leafceuticals, Hemp Inc., Calineva Farms, Happy Campers, Yield Farming, Franklin BioScience, Polaris MMJ, Acres, InPlanta Biotechnologies , Thompson Farm One, the Flowr Corporation and Green Harvest.
